# Mechanistic and Molecular Insights into Empagliflozin’s Role in Ferroptosis and Inflammation Trajectories in Acetaminophen-Induced Hepatotoxicity

**DOI:** 10.3390/ph18030405

**Published:** 2025-03-13

**Authors:** Aisha Alhaddad, Esraa M. Mosalam, Hind S. AboShabaan, Amany Said Sallam, Marwa M. Mahfouz, Enas Elhosary, Asmaa A. Mohammed, Ebtehal M. Metwally, Moataz A. Shaldam, Mai El-Sayed Ghoneim

**Affiliations:** 1 Department of Pharmacology and Toxicology, College of Pharmacy, Taibah University, Medina 42353, Saudi Arabia; aahaddad@taibahu.edu.sa; 2 Biochemistry Department, Faculty of Pharmacy, Menoufia University, Shebin El-Kom 32511, Menoufia, Egypt; 3 Department of Pharm D, Faculty of Pharmacy, Jadara University, Irbid 21110, Jordan; 4 Clinical Pathology Department, National Liver Institute Hospital, Menoufia University, Shebin El-Kom 32511, Menoufia, Egypt; drhindsaad@liver.menofia.edu.eg; 5 Department of Pharmacology and Toxicology, Faculty of Pharmacy, Menoufia University, Shebin El-Kom 32511, Menoufia, Egypt; amany.said@phrm.menofia.edu.eg (A.S.S.); marwamahfouz111@gmail.com (M.M.M.); 6 Department of Pathology, Faculty of Medicine, Helwan University, Cairo 11795, Egypt; enas.megahed@med.helwan.edu.eg; 7 Department of Pharmacology and Toxicology, Faculty of Pharmacy Girls, AL Azhar University, Cairo 11651, Egypt; asmaamahmoud.52@azhar.edu.eg; 8 Medical Physiology Department, Faculty of Medicine, Menoufia University, Shebin El-Kom 32511, Menoufia, Egypt; ibtehal.mitwali@med.menofia.edu.eg; 9 Department of Pharmaceutical Chemistry, Faculty of Pharmacy, Kafrelsheikh University, Kafrelsheikh 12613, Kafrelsheikh, Egypt; dr_moutaz_986@pharm.kfs.edu.eg; 10 Department of Pharmacy, “G. d’Annunzio” University of Chieti-Pescara, Via dei Vestini 31, 66100 Chieti, Italy; 11 Department of Pharmacology and Toxicology, Faculty of Pharmacy, University of Sadat City (USC), Sadat City 32897, Monufia Governorate, Egypt; mai.ghoneim@fop.usc.edu.eg

**Keywords:** acetaminophen, empagliflozin, ferroptosis, inflammation, oxidative stress, STAT3

## Abstract

**Background:** Acetaminophen (APAP)-induced acute liver injury (ALI) is increasingly becoming a public health issue with high rate of morbidity and mortality. Therefore, there is a critical demand for finding protective modalities by understanding the underlying proposed mechanisms including, but not limited to, ferroptosis and inflammation. **Objectives:** This study seeks to investigate the possible hepatoprotective effect of empagliflozin (EMPA) against APAP-induced ALI through modulation of ferroptosis and inflammatory cascades. **Methods:** Mice were allocated into the following five groups: vehicle control, APAP, EMPA 10, EMPA 20 (10 and 20 mg/kg/day, respectively, P.O.), and N-acetylcysteine (NAC, hepatoprotective agent against APAP-induced ALI). The hepatic injury was detected by determining liver enzymes and by histopathological examination. Inflammation, oxidative stress, apoptosis, and ferroptosis were also evaluated. **Results:** The APAP group showed an elevated level of hepatic enzymes with disrupted hepatic architecture. This toxicity was promoted by inflammation, oxidative stress, apoptosis, and ferroptosis, as indicated by elevated cytokines, lipid peroxidation, reduced antioxidants, increased caspase-3, decreased Bcl-2, and activation of the NF-κB/STAT3/hepcidin pathway. Pretreatment with EMPA remarkably reversed these features, which was reflected by restoration of the histoarchitecture of hepatic tissue, but the higher dose of EMPA was more efficient. **Conclusions:** APAP can induce ALI through initiation of inflammatory and oxidative conditions, which favor ferroptosis. EMPA hindered these unfavorable consequences; an outcome which indicates its anti-inflammatory, antioxidant, anti-apoptotic, and anti-ferroptotic effects. This modulatory action advocated EMPA as a potential hepatoprotective agent.

## 1. Introduction

Acetaminophen (APAP) is a widely used over the counter medication for relieving pain and fever. Unfortunately, APAP-induced acute liver injury (ALI) has emerged as a primary cause of mortality associated with drug-induced liver injury [1]. The hepatotoxicity of APAP is attributed to the formation of the toxic metabolite N-acetyl-p-benzoquinone imine (NAPQI), causing the depletion of glutathione (GSH) and overwhelming the antioxidant capacity of the cells [2]. While the pathogenesis of APAP-induced hepatotoxicity is multifaceted, it is unfortunate that N-acetylcysteine (NAC) is the only effective agent for treating APAP-induced ALI. Therefore, it is crucial to urgently identify new therapeutic modalities.

Ferroptosis is a distinct type of programmed cell death that involves an excessive accumulation of iron, diminished antioxidant capacity, and high reactive oxygen species (ROS) within the cells; resulting in oxidative cell damage [3]. Glutathione peroxidase 4 (GPX4) is a key regulator for the ferroptotic pathway, and acts by reducing lipid peroxides at the expense of GSH [4]. Several studies have demonstrated the significant impact of the accumulated iron within the cells, regulated by hepcidin, on the development of intracellular ferroptosis [5,6]. Interestingly, it has been found that reducing the levels of hepcidin had a positive influence on preventing ferroptosis [7]. Recently, ferroptosis has been identified as a culprit in the development of several liver ailments including non-alcoholic fatty liver disease [8], liver failure [9], hepatic ischemia/reperfusion [10], and APAP-induced hepatotoxicity [11]. More specifically, research has indicated that inhibitors of ferroptosis, including ferrostatin-1, substantially mitigate APAP-induced hepatic injury, lipid peroxidation, and mortality across multiple animal models [12,13]. The mechanism entails the depletion of GSH triggered by APAP toxicity, resulting in an imbalanced cellular redox status and accumulation of lipid peroxides [12]. Nevertheless, further exploration is necessary to determine the pivotal role of ferroptosis in APAP-induced ALI, which will provide an opportunity to create efficacious intervention approaches.

Empagliflozin (EMPA), a sodium-glucose cotransporter 2 (SGLT-2) inhibitor, is an antidiabetic drug that lowers blood sugar via decreasing glucose reabsorption in renal tubules [14]. In addition to its hypoglycemic effect, it possesses other beneficial therapeutic benefits, including nephroprotective, neuroprotective, and cardioprotective properties [15,16,17]. Moreover, it exhibited pleiotropic properties such antioxidant, anti-inflammatory, and antiapoptotic effects through the modulation of various molecular mechanisms [18]. Clinical studies have demonstrated the beneficial effects of EMPA on liver steatosis and fibrosis [19]. Additionally, experimental studies have shown its effectiveness against liver injuries induced by thioacetamide and ethanol [20,21]. However, its potential role in hepatotoxicity induced by APAP has not yet been investigated. Accordingly, the objective of this work was to delineate the possible hepatoprotective effect of EMPA against APAP-induced liver damage in experimental mice and to elucidate whether the ferroptosis signaling pathway is implicated in the development of liver injury triggered by APAP.

## 2. Results

### 2.1. EMPA Pretreatment Alleviated Liver Injury Induced by APAP

**Figure 1:**
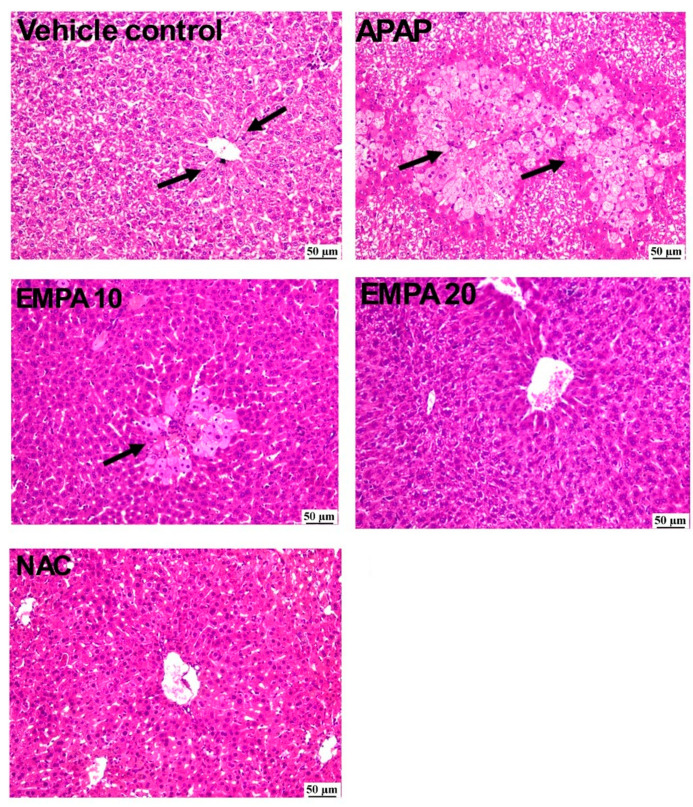
Effect of EMPA on histopathological changes in APAP-induced hepatotoxicity in mice (H&E). Photomicrographs of liver sections showing vehicle control group with normal hepatocytes in the centrilobular area (arrow). APAP showing marked hepatocellular degeneration and necrosis (arrows) in the centrilobular hepatocytes. EMPA 10 group with mild centrilobular hepatocellular necrosis (arrow). EMPA 20 group showing apparently normal hepatic parenchyma. NAC group with apparently normal hepatic parenchyma. APAP: acetaminophen; EMPA: empagliflozin; NAC: N-acetylcysteine.

Figure 1 demonstrates that the liver photomicrographs from the vehicle control group exhibited a histologically normal structure of hepatic parenchyma. In contrast, the sections from the APAP group showed severe confluent hepatocellular necrosis, especially in the centrilobular areas, with necrosis bridging the areas between the centrilobular zones. Moreover, clusters of mononuclear inflammatory cells were often found in many regions with hepatocellular necrosis. The group administered EMPA 10 showed a moderate degree of improvement, characterized by slight centrilobular hepatocellular damage but absence of bridging. Similarly, the group that was pretreated with the higher dose of EMPA exhibited a remarkable improvement with only occasional cell death identified in few parts, while other parts appeared to be unaffected. On the other hand, the photomicrographs of the NAC group displayed a notable recovery since the majority of the sections appeared to be nearly normal with only limited hepatocellular vacuolation. Regarding the hepatic lesion score, the APAP group exhibited a substantial high hepatic lesion score, which was significant with the normal group, while the EMPA 20-pretreated group showed a significant decrease in the lesion score compared to the diseased group. The groups can be arranged in a descendant manner as follows: APAP > EMPA 10 > EMPA 20 > NAC > vehicle control (Table 1).

**Figure 2:**
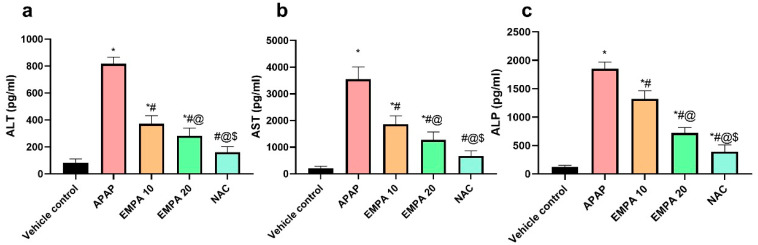
Effect of EMPA on liver enzymes in APAP-induced hepatotoxicity in mice. (**a**) ALT, (**b**) AST, and (**c**) ALP. The results are expressed as means ± SD (n = 6). The one-way ANOVA followed by the Tukey post hoc for multiple comparison were used to measure statistical significance. *p* < 0.05; * significance vs. vehicle control; # significance vs. APAP; @ significance vs. EMPA 10; $ significance vs. EMPA 20. ALT: alanine aminotransferase; AST: aspartate aminotransferase; ALP: alkaline phosphatase; APAP: acetaminophen; EMPA: empagliflozin; NAC: N-acetylcysteine.

Concerning the effect on hepatic enzymes, Figure 2 demonstrates a significant (*p* < 0.001) increase in the serum levels of ALT, AST, and ALP in the APAP group by 8.82, 15.97-, and 13.97-fold, respectively, as compared to the vehicle control. In contrast, EMPA pretreatment at doses of 10 and 20 mg/kg significantly reduced the serum levels of these enzymes by 54.49, 47.64, and 28.79%, respectively, for the lower dose, whereas reductions were by 74.8, 91.03, and 84.66%, respectively, for the higher dose. The NAC group also showed a remarkable recovery for hepatic enzymes, which was non-significant against vehicle control in the case of ALT (*p* = 0.074) and AST (*p* = 0.096), with a significant decline in the level of ALP (*p* = 0.002).

### 2.2. EMPA Pretreatment Restored the Imbalanced Redox State Induced by APAP

**Figure 3:**
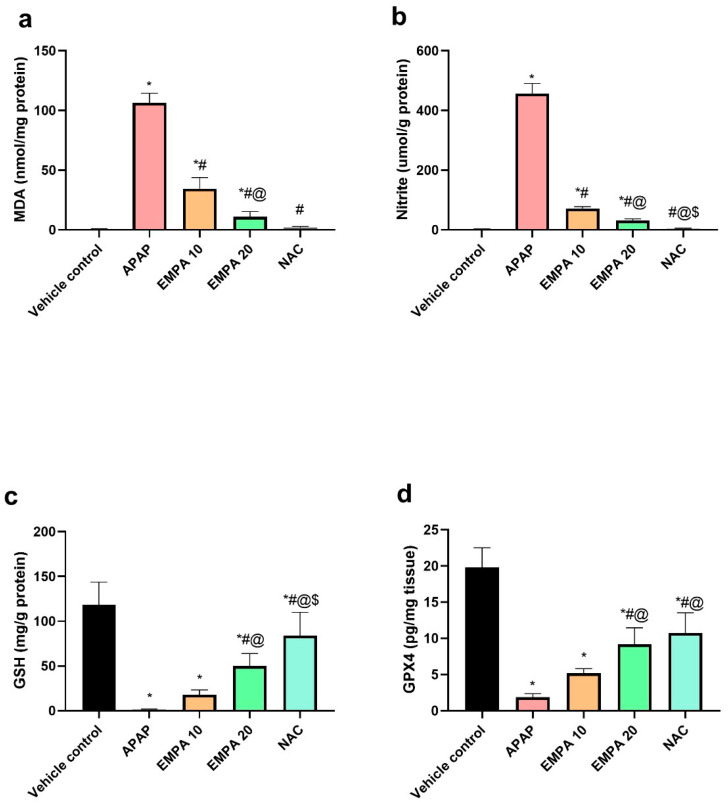
Effect of EMPA on oxidative stress in APAP-induced hepatotoxicity in mice. (**a**) MDA; (**b**) Nitrite; (**c**) GSH; (**d**) GPX4. The results are expressed as means ± SD (n = 6). The one-way ANOVA followed by the Tukey post hoc for multiple comparison were used to measure statistical significance. *p* < 0.05; * significance vs. vehicle control; # significance vs. APAP; @ significance vs. EMPA 10; $ significance vs. EMPA 20. APAP: acetaminophen; EMPA: empagliflozin; NAC: N-acetylcysteine; MDA: malondialdehyde; GSH: reduced glutathione;, GPX4: glutathione peroxidase.

Figure 3 shows that the hepatic contents of MDA and nitrite were significantly (*p* < 0.001) elevated in the APAP group in comparison with the vehicle control group. Furthermore, the APAP group showed a significant (*p* < 0.001) depletion in the hepatic GSH and GPX4 relative to the vehicle control group. On the contrary, the pretreatment with EMPA 10 showed a significant reduction in the level of MDA (67.49%, *p* < 0.001) and nitrite (84.29%, *p* < 0.001), along with a remarkable rise in the hepatic GSH (12.25-fold, *p* = 0.486) and GPX4 (1.74-fold, *p* < 0.001) compared to the APAP group. Likewise, the higher dose of EMPA showed similar findings with significant reversal effects on these biomarkers (89.72%, 93.21-, and 35.81-fold, and 3.87-fold, respectively, for MDA, nitrite, GSH, and GPX4) relative to the diseased group. It is worth noting that NAC group also exhibited a significant (*p* < 0.001) decrease in MDA and nitrite, together with a significant rise in the level of GSH and GPX4 compared to the APAP group.

### 2.3. EMPA Pretreatment Counteracted Hepatic Inflammation Induced by APAP

**Figure 4:**
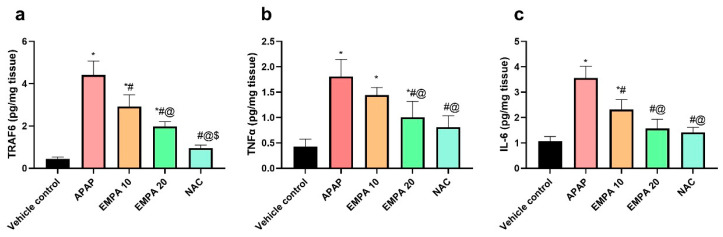
Effect of EMPA on inflammatory mediators in APAP-induced hepatotoxicity in mice. (**a**) TRAF 6, (**b**) IL-6, and (**c**) TNF-α. The results are expressed as means ± SD (n = 6). The one-way ANOVA followed by the Tukey post hoc for multiple comparison were used to measure statistical significance. *p* < 0.05, * significance vs. vehicle control, # significance vs. APAP, @ significance vs. EMPA 10, $ significance vs. EMPA 20. APAP: acetaminophen, EMPA: empagliflozin; NAC: N-acetylcysteine; TRAF6: tumor necrosis factor receptor-associated factor 6; TNF-α: tumor necrosis factor alpha; IL-6: interleukin 6.

As shown in Figure 4, the APAP mice showed a significant (*p* < 0.001) increase in TRAF 6, IL-6, and TNF-α by 8.97-, 2.34-, and 3.24-fold, respectively, in comparison with the normal mice. Protection of the mice with EMPA 10 reversed the level of these inflammatory mediators by 33.76 (*p* < 0.001), 34.99% (*p* < 0.001), and 20.25 (*p* = 0.108), respectively, when compared to the APAP mice. Similarly, EMPA 20 diminished these biomarkers significantly (*p* < 0.001) compared to the diseased group by 55.03, 55.95, and 44.4%, correspondingly, which was superior to the lower dose. The NAC-treated mice showed a favorable effect on TRAF 6, IL-6, and TNF-α, which was comparable and non-significant with the normal mice with the exception of TRAF6.

**Figure 5:**
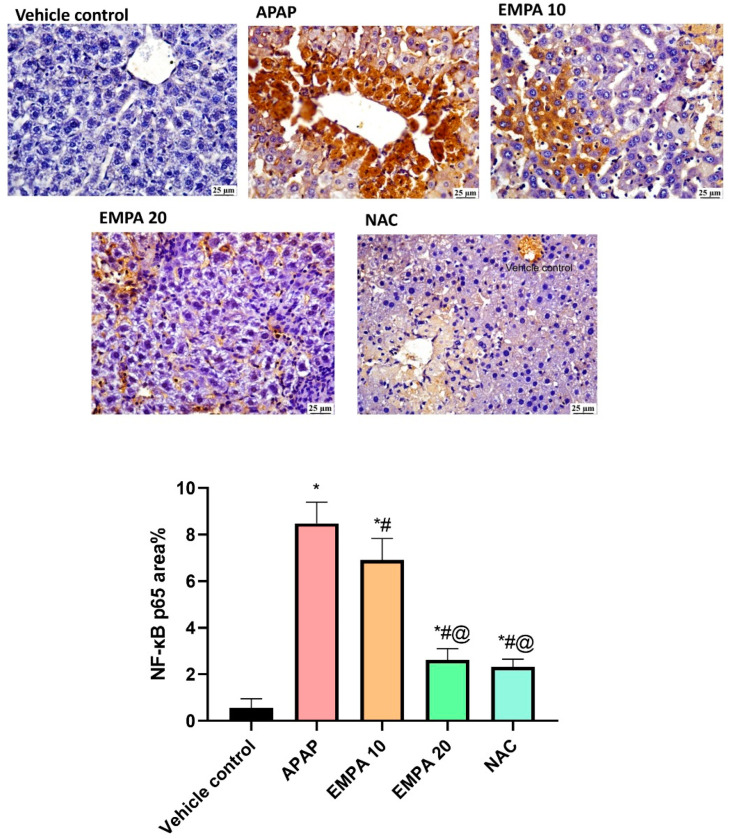
Effect of EMPA on immuno-staining of NF-қB in APAP-induced hepatotoxicity in mice. The results are expressed as mean ± SD (n = 5). The one-way ANOVA followed by the Tukey post hoc for multiple comparison were used to measure statistical significance. *p* < 0.05, * significance vs. vehicle control, # significance vs. APAP, @ significance vs. EMPA 10. APAP: acetaminophen; EMPA: empagliflozin; NAC: N-acetylcysteine; NF-κB p65: nuclear factor kappa B subunit 65.

Regarding the effect on NF-қB, there was a strong immuno-reactivity toward anti-NF-қB in the APAP group. A moderate reactivity was detected in EMPA 10-pretreated mice, while mild reactivity was noticed in EMPA 20 and NAC groups. In a quantitative measure, there was a significant (*p* < 0.001) increase in the level of this transcription factor by 14.25-fold in APAP group compared to normal. In contrast, the mice that were protected with EMPA, in both doses 10 and 20, showed a significant reduction in the expression level of NF-қB by 18.55 (*p* = 0.009) and 68.97% (*p* < 0.001), respectively, compared to the untreated group. The same effect was observed in NAC-treated mice as displayed in Figure 5.

### 2.4. EMPA Pretreatment Reduced Apoptotic Changes in Liver Induced by APAP

**Figure 6:**
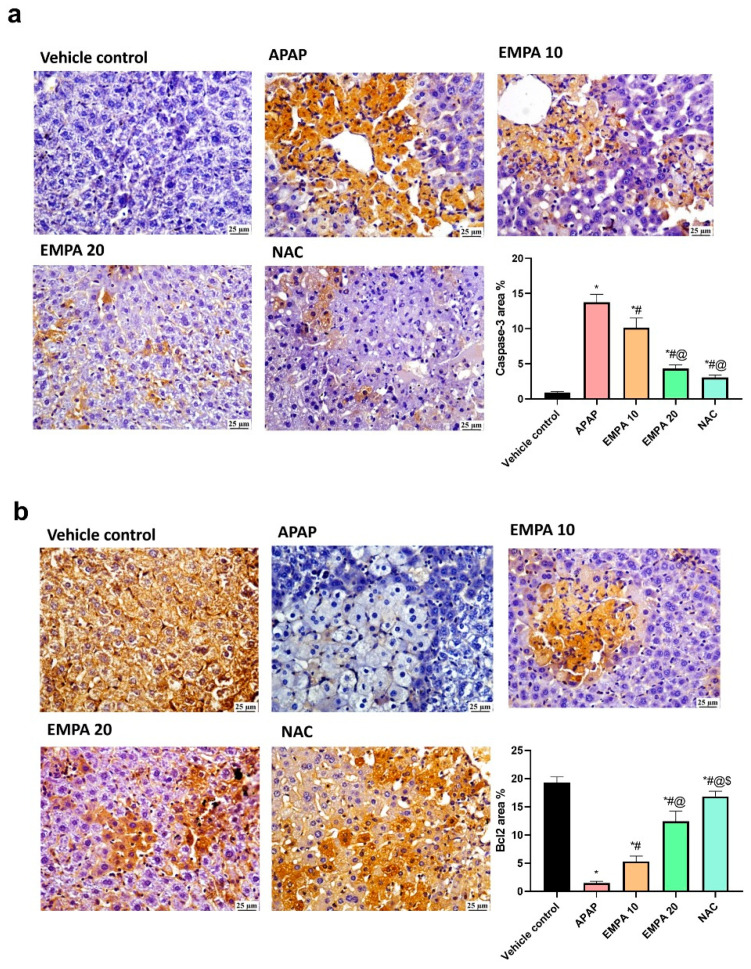
Effect of EMPA on apoptosis in APAP-induced hepatotoxicity in mice. (**a**) represents photomicrograph of livers of the studied groups and chart quantification as area percentage of caspase-3 immunoexpression; (**b**) represents photomicrographs of livers of the studied groups and chart quantification as area percentage of Bcl2 immunoexpression. The results are expressed as mean ± SD (n = 5). The one-way ANOVA followed by the Tukey post hoc for multiple comparison were used to measure statistical significance. *p* < 0.05; * significance vs. vehicle control; # significance vs. APAP; @ significance vs. EMPA 10; $ significance vs. EMPA 20. APAP: acetaminophen; EMPA: empagliflozin; NAC: N-acetylcysteine; Bcl2: B-cell lymphoma 2.

Figure 6 shows that the induction of ALI by APAP significantly (*p* < 0.001) increased the reactivity of caspase-3 toward its specific antibody by 13.71% relative to the normal group. The opposite effect was observed for the anti-apoptotic Bcl2, where it was decreased significantly (*p* < 0.001) by 92.42%. Protection of the mice with EMPA 10 and 20 reversed these findings (↓ caspase-3 by 26.17 and 68.58%, respectively, *p* < 0.001, whereas ↑ Bcl2 by 2.66- and 7.52-fold, *p* < 0.001, respectively). NAC group also decreased the reactivity of caspase-3 and increased that of Bcl2 relatively to the untreated group.

### 2.5. EMPA Pretreatment Diminished Liver Iron Deposition-Evoked Ferroptosis Induced by APAP

**Figure 7:**
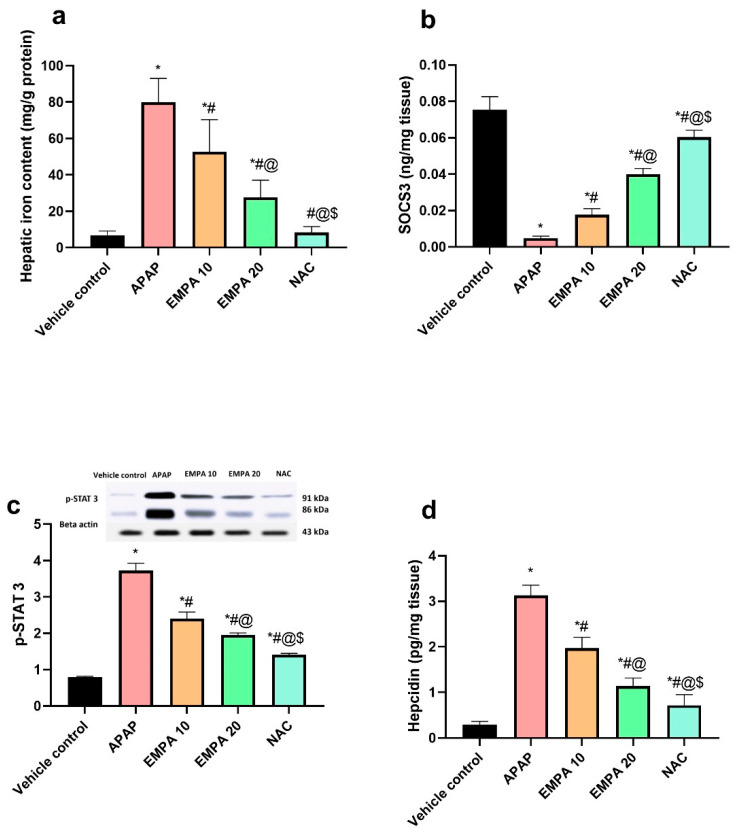
Effect of EMPA on ferroptosis in APAP-induced hepatotoxicity in mice. (**a**) hepatic iron content; (**b**) SOCS3; (**c**) p-STAT3; and (**d**) hepcidin. The results are expressed as means ± SD of 6 mice, but only 3 for p-STAT3. The one-way ANOVA followed by the Tukey post hoc for multiple comparison were used to measure statistical significance. *p* < 0.05; * significance vs. vehicle control; # significance vs. APAP; @ significance vs. EMPA 10; $ significance vs. EMPA 20. APAP: acetaminophen; EMPA: empagliflozin; NAC: N-acetylcysteine; SOCS3: suppressor of cytokine signaling 3; p-STAT3: phosphorylated signal transducers and activators of transcription 3.

As shown in Figure 7, the APAP group significantly (*p* < 0.001) raised the hepatic iron content (10.5-fold), p-STAT3 (3.69-fold), and hepcidin (9.7-fold) along with a significant (*p* < 0.001) decrease in SOCS3 (93.59%) compared to the normal group. Comparing with the diseased mice, pretreatment with EMPA 10 and 20 showed a significant (*p* < 0.01) reversal pattern of these biomarkers. EMPA 10 decreased the iron load (33.87%), p-STAT3 (35.54%), and hepcidin (37.17%) with an increase in SOCS3 (2.68-fold). Similar outcomes were observed with EMPA 20 but they were more superior than the lower dose (↓ 65.41%, ↓ 47.53%, ↓ 63.75%, and ↑ 7.29-fold, respectively for the hepatic iron, p-STAT3, hepcidin, and SOCS3. NAC also showed the same pattern of the results as EMPA-treated groups.

### 2.6. Protein–Protein Interaction (PPI) Network

**Figure 8:**
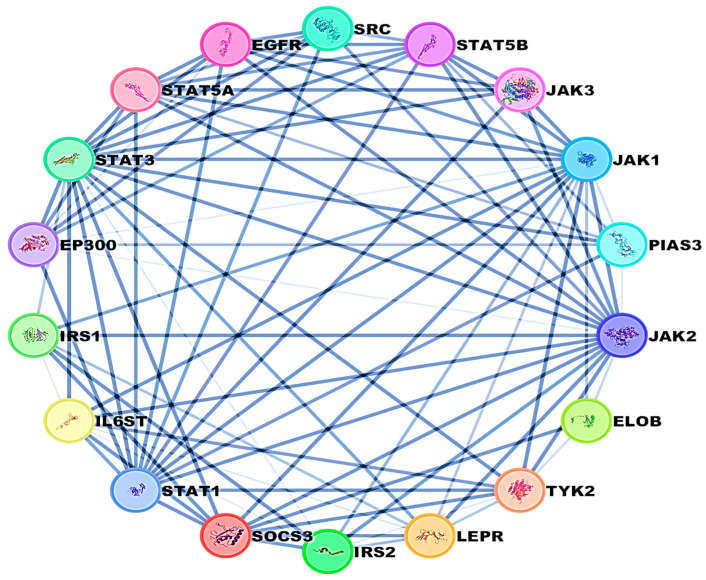
Protein–protein interaction network between SOCS3, STAT3, and other target molecules.

A combined network analysis was conducted between SOCS3 and STAT3 to identify associated molecules that could serve as potential targets for liver injury. The network demonstrated interactions between these two proteins with other pathways, as illustrated in Figure 8.

### 2.7. Docking of EMPA

**Figure 9:**
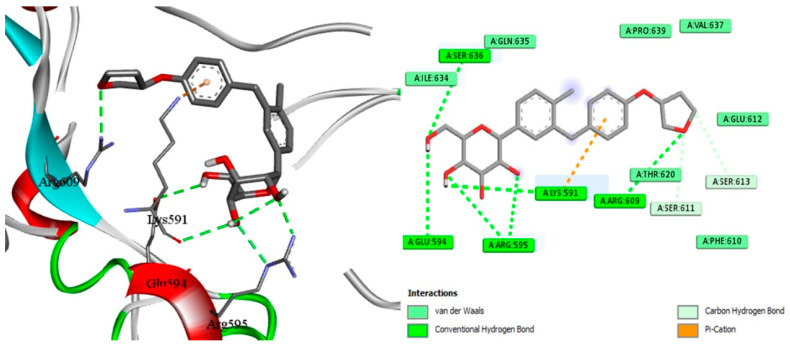
Docking of EMPA inside the binding pocket of STAT3.

**Figure 10:**
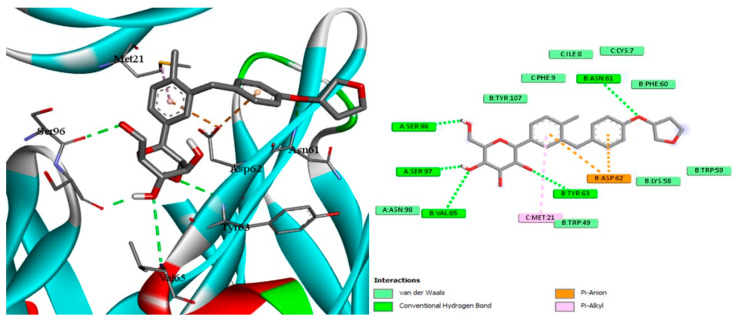
Docking of EMPA inside the binding pocket of hepcidin.

**Figure 11:**
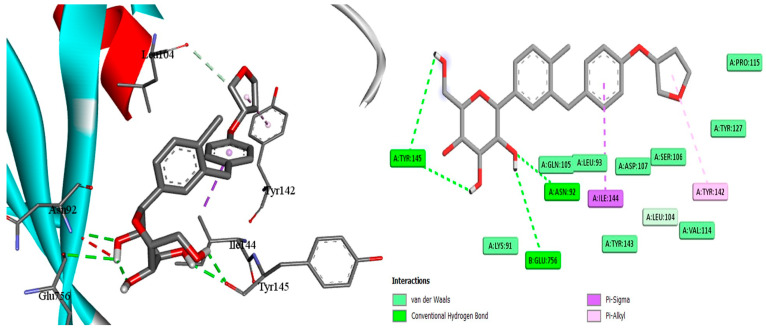
Docking of EMPA inside the binding pocket of SOC3.

With the aid of the docking tool AutoDock Vina version 1.1.2, molecular docking with grid-based energy assessment was carried out to examine the binding behavior of empagliflozin towards hepcidin, STAT3, and SOCS3. After calculating binding free energy, the order of STAT3 (−7.5 kcal/mol) > hepcidin (−7.2 kcal/mol) > SOCS3 (−6.5 kcal/mol) for empagliflozin binding was determined. Empagliflozin was well-accommodated in the binding pocket of STAT3 with six hydrogen bonds with Lys591, Glu594, Arg595, Thr620, and Ser636, which contribute to the high binding energy between STAT3 and empagliflozin (Figure 9). Along with other hydrophobic interactions, a π–cation contact between the phenyl ring of empagliflozin and the Lys591 basic group was also noted. Likewise, empagliflozin reaches hepcidin’s binding pocket with high affinity and interacts with the target through π interactions, hydrogen bonds, and hydrophobic contacts (Figure 9). Empagliflozin binds Asn61, Tyr63, Val65, Ser96, and Ser97 in five hydrogen bonds. Furthermore, through its aromatic rings, it forms a different π anion with Asp62 in addition to different hydrophobic interactions. Moreover, empagliflozin has a good binding affinity upon entering SOC3′s binding pocket, and it engages in a variety of interactions including hydrogen bond and van der Waals interactions (Figure 10). Four hydrogen bonds are formed between Asn92, Tyr145, and Glu756 by empagliflozin. Along with other hydrophobic contacts, it also establishes a π–sigma interaction with Ile144 through its aromatic phenyl ring (Figure 11). Ultimately, these interactions support empagliflozin’s moderate to high affinity for the three targets.

## 3. Discussion

The present study aimed to explore the hepatoprotective effect of EMPA in APAP-induced liver injury and elucidate the possible underlying mechanism through investigating the ferroptosis signaling. The findings indicated that EMPA can improve the APAP-induced alterations in biochemical parameters (AST, ALT, and ALP), histopathological architecture, oxidative stress biomarkers (MDA, nitrite, GSH, and GPX4), as well as suppressing inflammation, apoptosis, and ferroptosis. Indeed, this work is the first to show that EMPA impeded APAP-induced hepatotoxicity by inhibiting IL-6/STAT3/hepcidin and NF-κB axis, which significantly contribute to the induction of ferroptosis.

In our investigation, APAP-induced liver injury was characterized by an increased level of serum hepatic enzymes, and alterations in the liver histoarchitecture. These modifications indicate the damage caused to hepatocytes, which is consistent with prior findings that have revealed the catastrophic effect of APAP on the liver [22,23]. On the contrary, pre-administration of EMPA reversed these abnormalities with subsequent amendment of liver functions and cellularity. Our findings are in alignment with studies that have shown EMPA’s positive influence on liver tissue in various disease models [20,21,24], and thereby, confirming its hepatoprotective effect.

Ample evidence has indicated that iron overload and oxidative stress have been identified as crucial factors contributing to the induction of ferroptosis in several liver paradigms [25,26], including APAP-induced liver injury [11,27]. Mechanistically, excessive iron accumulation in the cell interacts with hydrogen peroxide via a Fenton reaction, which leads to the lipid peroxidation of polyunsaturated fatty acid and ultimately initiating ferroptosis [28,29,30]. Notably, GPX4, a key regulatory factor of ferroptosis, reduces lipid hydroperoxides using GSH as a reducing cofactor and its blockade could induce the ferroptosis pathway [28]. Consistent with these outcomes, our study documented that the induction of ferroptosis and oxidative stress was evidenced by elevated hepatic iron content, MDA, and nitrite, along with reduced levels of endogenous antioxidants such as GSH and GPX4 in APAP-treated mice. These findings reflect an imbalanced redox status in the hepatic tissue and ferroptosis stimulation, which aligns with several prior reports [31,32].

Nevertheless, EMPA pretreatment effectively reversed the changes induced by APAP in terms of hepatic iron content and oxidative stress; thus, reinforcing the anti-ferroptotic effect of EMPA, as reported in earlier studies [33,34]. Additionally, a recent study has elucidated that EMPA effectively inhibits cyclic stretch-induced ROS production in endothelial cells in the coronary artery by blocking protein kinase activity [35]. Furthermore, previous research has reported that EMPA boosts the effectiveness of antioxidants in combating lipo-toxicity and attenuates ferroptosis in diabetic kidney disease by activating Nrf2 and AMPK pathways [34,36]. Consequently, these outcomes highlight the antioxidant and anti-ferroptotic effects of EMPA against liver injury.

A plethora of studies have demonstrated the significant involvement of the NF-κB pathway in the development of APAP-induced ALI [23,37,38]. Activation of NF-κB induces the transcription of proinflammatory mediators, including IL-6 and TNF-α, which exacerbates the liver injury [39,40]. Furthermore, the NF-κB pathway plays a crucial role in triggering oxidative stress; hence, producing a vicious cycle of oxidative stress and inflammation [41]. Interestingly, several reports have figured out the correlation between NF-κB activation and induction of ferroptosis [42,43,44]. Aligning with these observations, our results demonstrated that APAP upsurged TRAF 6 expression and consequently led to an increase in the level of NF-κB, leading to the production of proinflammatory mediators, which ultimately caused hepatic damage. However, EMPA pretreatment significantly reduced the levels of these parameters, and this was accompanied by an upregulation of GPX4. These finding are in accordance with the previous research indicating that EMPA effectively reduced inflammation through shutting down of the NF-κB pathway in several disease models such as ulcerative colitis [45] and streptozotocin-induced diabetes [46]. Collectively, the anti-inflammatory effect of EMPA was partly the cause behind the inhibitory effect on TRAF6/NF-κB pathway.

More importantly, the activation of the STAT signaling pathway plays an important role in the pathophysiology of APAP-induced hepatotoxicity [47,48]. Binding of IL-6 and TNF-α to their respective receptors triggers hepatic STAT3 phosphorylation, followed by nuclear translocation, and finally the activation of the downstream signaling pathways. Prior research demonstrated that hepcidin expression is stimulated by IL-6-mediated hepatic STAT3 activation [44,49]. It has been also discovered that an increased level of hepcidin can hinder the liver’s ability to release iron by breakdown of ferroportin, a key transporter of cellular iron, leading to a significant iron deposition [50,51]. Furthermore, several studies have revealed that a reduced level of hepcidin may have a beneficial impact on preventing ferroptosis in various disease models, such as subarachnoid hemorrhage [7], chronic atrophic gastritis [52], and acute liver injury [53,54,55]. Hence, the strategy of targeting the IL-6/STAT3/hepcidin axis has demonstrated considerable promise for mitigating ferroptosis in various disease models [8,56,57]. In accordance with these findings, we observed that APAP elevated IL-6 and p-STAT3, with an increased level of hepcidin. On the other hand, EMPA pretreatment reversed these clues. Comparable results showed that EMPA could abate the expression of STAT3 in various scenarios, including lipopolysaccharide-induced inflammation [58] and autoimmune myocarditis [59]. Furthermore, Ali et al. (2022) showed that EMPA ameliorates type II diabetes in rats by decreasing hepcidin level [60]. Accordingly, EMPA can be a potent anti-ferroptotic through targeting the IL-6/STAT3/hepcidin axis.

It is worth noting that SOCS3 plays a pivotal role in inhibiting STAT3 signaling, which, in turn, suppresses Janus kinase (JAK) phosphorylation and consequently limits STAT3 phosphorylation [57]. In the present investigation, the reduced SOCS3 level caused by APAP was dramatically increased with EMPA pretreatment. Although this is the first study to show how EMPA modulates the expression of SOCS3, there is mounting evidence that can support our findings such as controlling NF-κB and IL-6/STAT3/SOCS3 hubs in an acute kidney damage model [61], suppressing the NF-κB/STAT3/SOCS3 axis in experimental asthma [62], and modifying IL-6/JAK/STAT3/SOCS3 signaling in testicular injury [63].

Apoptosis, another type of programmed cell death, plays a crucial role in APAP-induced ALI [23,64,65]. It was found that pro-inflammatory cytokines and NF-κB can trigger apoptotic cell death via stimulating the caspase family [66]. In accordance with this notion, the hepatic injury induced by APAP was also mediated through apoptosis, as evidenced by upregulated caspase-3, and downregulated anti-apoptotic Bcl-2, which is consistent with previous studies [67,68]. Remarkably, excessive production of ROS has been found to trigger apoptosis and pave the way for induction of ferroptosis [69]. Notwithstanding, these hallmarks were amended after the administration of EMPA. Our results are consistent with the preceding studies demonstrating that EMPA has the ability to halt apoptosis in ethanol-induced cardiac injury [70] and also in a kidney injury model [71]. These data provide evidence that EMPA exerts potential anti-inflammatory and anti-apoptotic effects, which further hinders ferroptosis.

Docking simulation and PPI network analyses were also conducted to provide more mechanistic insights that support our main findings regarding the protective effect of EMPA against APAP-induced liver injury. The PPI network highlights the central role of STAT3 and SOCS3 in liver inflammation and also shows their interactions with other key regulatory proteins and pathways. Furthermore, molecular docking simulations reveal the direct binding interactions between EMPA and each of STAT3, hepcidin, and SOCS3; suggesting a potential mechanism by which EMPA modulates these trajectories. These findings support the aim of the current work that EMPA may exert hepatoprotective effects by modulating the STAT3/SOCS3 axis and iron homeostasis through hepcidin regulation.

## 4. Materials and Methods

### 4.1. Chemicals and Reagents

APAP was obtained as 10 mg/mL solution for i.v. infusion from ARABCOMED (Cairo, Egypt). EMPA was obtained commercially as tablets from Zeta Pharma^®^ (Cairo, Egypt). NAC 600 mg granules were purchased commercially from SEDICO (Cairo, Egypt). Other chemicals were of high grade and quality.

### 4.2. Experimental Animals

Thirty male albino mice, weighing 30–35 g, were purchased from VACSERA (Cairo, Egypt). The mice were placed in suitable cages supplied with tap water and commercial food ad libitum and they were exposed to normal cycles of day and night. The current study was approved by the Scientific Research Ethical Committee, Faculty of Pharmacy, Menoufia University (approval number: MPIR 23/01).

### 4.3. Study Design

In a random manner, the mice were divided into five different groups (n = 6), as follows: vehicle control, APAP, EMPA 10, EMPA 20, and NAC. Hepatotoxicity was induced by a single dose (300 mg/kg, i.p.) of APAP in the seventh day of the experiment [72] for all groups except for the vehicle control. EMPA was administered at a dose of 10 or 20 mg/kg via oral route for 7 successive days [73], and on the last day, APAP was administered one hour after the last dose. The vehicle control group was receiving normal saline.

### 4.4. Collection of the Samples

The mice were sacrificed under light halothane anesthesia at the end of the experiment. The blood samples were gathered in plane tubes to separate the serum by centrifugation. The liver samples were cut off, rinsed, and split into small parts for the biological assays and were kept in −80 °C. Other parts of these samples were preserved in 10% formalin for histopathological examination and immunohistochemical investigation.

### 4.5. Histopathology and Lesion Score

Five liver samples from each group were kept in neutral buffered formalin (10%) for fixation followed by routine processing protocol in different grades of alcohol and xylene ending with embedding in paraffin wax. Five µm sections were sliced and stained with hematoxylin and eosin (H&E) for light microscopy [74]. For evaluation of liver injury, a score from 0 to 3 was given for each studied group as follows: 0 = normal liver sections; 1 = slight congestion and necrosis of solitary hepatocytes, limited to centrilobular area, many of the lobules not affected; 2 = reasonable congestion and hemorrhage of the zones around the centrilobular vein and spreading into the midzonal cells, most lobules are affected; additionally, zones of confluent necrosis restrained to the hepatocytes surrounding the centrilobular vein; and 3 = prevalent parts of congestion and hemorrhage in the centrilobular and midzonal areas of the liver. Confluent coagulative necrosis affecting nearly all hepatocytes in the centrilobular zone; bridging of spots of necrosis between centrilobular zones is common [75].

### 4.6. Immunohistochemistry

Five liver samples from each group were cut on adhesive slides, deparaffinized, and rehydrated. Afterward, a heat-induced epitope retrieval step was conducted, and tissue sections were incubated with primary anti-caspase-3, NF-қB (at a dilution of 1:200, Sanat Cruz Biotechnology, Dallas, TX, USA), and BCl-2 (at a dilution of 1:200, Proteintech Group, Inc., Rosemont, IL, USA) for an hour at room temperature. After washing, an HRP-labelled detection kit (Bio SB, Goleta, CA, USA) was used as specified by the manufacturer’s instructions. Negative control was obtained by excluding incubation with the primary antibodies. Positive expression was quantified as area percent in five random microscopic fields representing each group.

### 4.7. Determination of Liver Toxicity Parameters

The levels of alanine aminotransferase (ALT, Cat no. ab282882), aspartate aminotransferase (AST, Cat no. ab263882), and alkaline phosphatase (ALP, Cat no. ab285274) were determined in the serum by commercially available ELISA kit purchased from Abcam (Waltham, MA, USA) according to the supplier’s instructions.

### 4.8. Determination of Oxidative Stress Markers

The hepatic content of malondialdehyde (MDA, Cat no. E-BC-K025-S), nitrite (Cat No. E-BC-K070-S), and GSH (Cat no. E-BC-K030-S) was determined in the liver tissue homogenate, as specified by commercial colorimetric kits purchased from Elabscience^®^ (Houston, TX, USA). Glutathione peroxidase 4 (GPX4, Cat no. A303448) was also determined in the tissue homogenate as stated in a specific ELISA kit brought from Antibodies (St. Louis, MO, USA).

### 4.9. Determination of Inflammatory Markers

Concentration of TRAF 6 (Cat no. LS-F53451, LifeSpan BioSciences, Shirley, MA, USA), IL-6 (Cat no. E-EL-M0044, Elabscience^®^, Houston, TX, USA), and TNF-α (Cat no. E-EL-M3063, Elabscience^®^, Houston, TX, USA) was estimated in the liver homogenized samples by commercial ELISA kits according to the manufacturer’s instructions.

### 4.10. Determination of STAT3 by Western Blot Assay

Three samples from each group were selected for Western Blot analysis. At the start, the target protein was obtained from the tissue by ReadyPrep^TM^ protein extraction kit (Bio-Rad, Contra Costa County, CA, USA). The extracted protein was then quantified by Bradford Protein Assay Kit (BIO BASIC, Markham, ON, Canada). Afterward, 20 μg of the extracted protein was mixed up equally with 2× Laemmli sample buffer and the protein was separated using SDS-PAGE electrophoresis system (Bio-Rad, Contra Costa County, CA, USA). Then, the protein was transferred to a membrane using Trans-Blot Turbo transfer system (Bio-Rad, Contra Costa County, CA, USA). The resulting bands were visualized and imaged using stain-free blot technology by an imaging system. The membrane was blockaded by tris-buffered saline with 0.1% tween (TBST) and 3% bovine serum albumin (BSA). The target protein was then incubated with anti-STAT3 antibody (Sanat Cruz Biotechnology, TX, USA) followed by washing and incubation with goat anti-rabbit IgG secondary antibody [HRP] (Novus Biologicals, Centennial, CO, USA). The chemiluminescent Clarity Western ECL substrate (Bio-Rad, CA, USA) was applied to the blots as specified by the supplier. The signals were caught using a CCD camera-based imager. ChemiDoc MP imaging system (Bio-Rad, Contra Costa County, CA, USA) was used to estimate the band intensity against β-actin reference gene.

### 4.11. Determination of Ferroptosis-Related Biomarkers

Concentration of SOCS 3 (Cat no. LS-F68453) and hepcidin (Cat no. LS-F11620) was determined in the liver homogenate by using specific ELISA kits purchased from LifeSpan BioSciences (Shirley, MA, USA) and the assays were conducted consistent with the supplier’s instructions. Moreover, the concentration of iron was also determined in the tissue homogenate using a colorimetric method (Cat no. E-BC-K139-M, Elabscience^®^, Houston, TX, USA).

### 4.12. PPI Network Analysis

SOCS3 and STAT3 were selected as key proteins to establish a PPI network for predicting additional molecular and downstream targets. The protein network was constructed using Cytoscape software version 3.10.3.

### 4.13. Experimental Docking

For molecular docking studies, the protein data bank was utilized to obtain STAT3 (PDB: 1BG1) [76], hepcidin (PDB: 3H03) [77], and SOC3 (PDB: 2HMH) [78]. The co-crystallized ligand and empagliflozin were used in docking research. The docking was performed using AutoDock Vina, version 1.1.2 [79]. Using the AutoDockTools program, version 1.5.7 [80], the pdbqt files for the receptors were made by removing water molecules, adding hydrogen, and then using the Gasteiger method [81] to calculate the atomic partial charges. For the docking, the exhaustiveness and num modes were set to 10 and 14, respectively. The grid box with coordinates of (x = 106.9, y = 76.9, and z = 60.1) with a size of (x = 38.4, y = 22.7, z = 30.3) was shown to be the active site for STAT3. The grid box with coordinates of (x = −44.5, y = 24.8, and z = 11.15) and size of (x = 38.4, y = 22.7, and z = 30.3) was shown to be the active site for hepcidin. The grid box with coordinates of (x = 16.6, y = 6.0, and z = 43.5) with a size of (x = 22.0, y = 22.0, and z = 22.0) was found to be the active location for SOC3. The 3D visualization was constructed using Biovia Discovery Studio 2023 Client version 2023.

### 4.14. Statistical Analysis

Data were assessed by the Statistical Package for Social Sciences, SPSS (IBM, Armonk, NY, USA), version 22.0 software. The results were displayed as the mean ± standard deviation (SD). One-way analysis of variance (ANOVA) followed by Tukey post hoc test were used for statistical analysis. The Kruskal–Wallis one-way ANOVA (K samples) with pairwise multiple comparisons test was used for nonparametric data and those data were expressed as median with the minimum and the maximum values. *p* < 0.05 was considered statistically significant.

## 5. Conclusions

**Figure 12:**
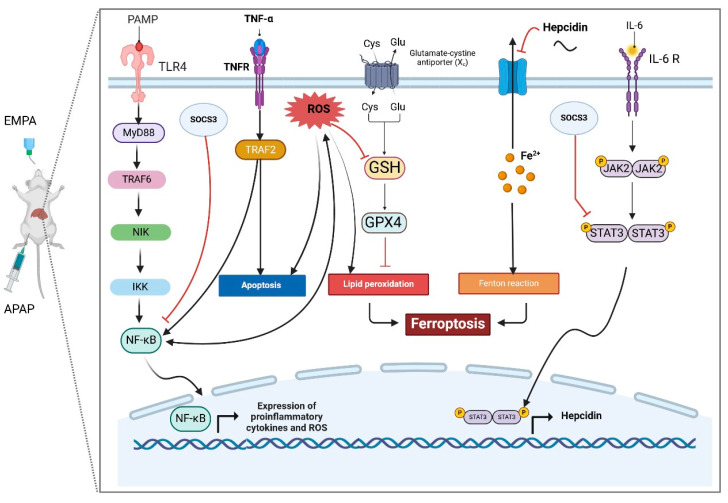
Illustration for the ferroptosis signaling and molecular mechanism of EMPA.

APAP-induced hepatotoxicity can be driven by induction of inflammation and oxidative stress with subsequent activation of ferroptosis through triggering NF-κB/STAT3/hepcidin signaling machinery. Administration of EMPA hindered these unfavorable consequences; the outcome of which indicates its anti-inflammatory, antioxidant, anti-apoptotic, and anti-ferroptotic effects (Figure 12). This modulatory action advocated EMPA as a potential hepatoprotective agent. However, further investigations and clinical trials are highly recommended to expand our findings.

## Figures and Tables

**Table 1:** Effect of EMPA on lesion score of the liver.

Groups	Median	Min	Max
Vehicle control	0	0	0
APAP	3 *	2	3
EMPA 10	2 *	1	2
EMPA 20	1 ^#^	0	1
NAC	0 ^#, @^	0	1

Kruskal–Wallis one-way ANOVA (K samples) with pairwise multiple comparisons test was used (n = 5). *p* < 0.05, * significance vs. vehicle control, # significance vs. APAP, @ significance vs. EMPA 10. APAP: acetaminophen, EMPA: empagliflozin, NAC: N-acetylcysteine.

## Data Availability

Data will be available upon reasonable request from authors for privacy reasons.
